# *Dec1* Deficiency Suppresses Cardiac Perivascular Fibrosis Induced by Transverse Aortic Constriction

**DOI:** 10.3390/ijms20194967

**Published:** 2019-10-08

**Authors:** Hue Thi Le, Fuyuki Sato, Akira Kohsaka, Ujjal K. Bhawal, Tomomi Nakao, Yasuteru Muragaki, Masanori Nakata

**Affiliations:** 1 Department of Physiology, Wakayama Medical University, Wakayama 641-8509, Japan; huele23989@gmail.com (H.T.L.); kohsaka@flamenco.plala.or.jp (A.K.); mnakata@wakayama-med.ac.jp (M.N.); 2 Department of Physiology, Hanoi Medical University, Hanoi 100000, Vietnam; 3 Department of Pathology, Wakayama Medical University, Wakayama 641-8509, Japan; ymuragak@wakayama-med.ac.jp; 4 Department of Biochemistry and Molecular Biology, Nihon University School of Dentistry at Matsudo, Matsudo 271-8587, Japan; bhawal.ujjal.kumar@nihon-u.ac.jp

**Keywords:** *Dec1*, cardiac fibrosis, immunohistochemistry, αSMA, S100A4

## Abstract

Cardiac fibrosis is a major cause of cardiac dysfunction in hypertrophic hearts. *Differentiated embryonic chondrocyte gene 1* (*Dec1*), a basic helix–loop–helix transcription factor, has circadian expression in the heart; however, its role in cardiac diseases remains unknown. Therefore, using *Dec1* knock-out (*Dec1*KO) and wild-type (WT) mice, we evaluated cardiac function and morphology at one and four weeks after transverse aortic constriction (TAC) or sham surgery. We found that *Dec1*KO mice retained cardiac function until four weeks after TAC. *Dec1*KO mice also revealed more severely hypertrophic hearts than WT mice at four weeks after TAC, whereas no significant change was observed at one week. An increase in *Dec1* expression was found in myocardial and stromal cells of TAC-treated WT mice. In addition, *Dec1* circadian expression was disrupted in the heart of TAC-treated WT mice. Cardiac perivascular fibrosis was suppressed in TAC-treated *Dec1*KO mice, with positive immunostaining of S100 calcium binding protein A4 (S100A4), alpha smooth muscle actin (αSMA), transforming growth factor beta 1 (TGFβ1), phosphorylation of Smad family member 3 (pSmad3), tumor necrosis factor alpha (TNFα), and cyclin-interacting protein 1 (p21). Furthermore, *Dec1* expression was increased in myocardial hypertrophy and myocardial infarction of autopsy cases. Taken together, our results indicate that *Dec1* deficiency suppresses cardiac fibrosis, preserving cardiac function in hypertrophic hearts. We suggest that *Dec1* could be a new therapeutic target in cardiac fibrosis.

## 1. Introduction

Left ventricular pressure overload induced by transverse aortic constriction (TAC) reportedly triggers inflammation and hypoxia in the heart [1,2,3,4]. Previous reports showed that cardiac hypertrophy had an adaptive response to maintain cardiac function at one week after TAC in mice [4,5]. Cardiac fibrosis was reported as a major feature of ventricular remodeling in hypertrophic hearts, leading to cardiac dysfunction at four weeks after TAC in mice [4,5,6]. In hypertrophic hearts, inflammation was shown to play an important role in the development of cardiac fibrosis [7,8].

Mice treated by TAC showed an increase in fibrosis, involving S100 calcium binding protein A4 (S100A4), alpha smooth muscle actin (αSMA), transforming growth factor beta 1 (TGFβ1), phosphorylation of Smad family member 3 (pSmad3), tumor necrosis factor alpha (TNFα), and cyclin-interacting protein 1 (p21) [2,9,10]. S100A4, also known as fibroblast-specific protein 1 (FSP1), was suggested as a marker of fibrosis [11]. In addition, αSMA is a marker of smooth muscle cells and myofibroblasts [12]. S100A4 and αSMA had little expression in fibroblasts under normal conditions, whereas their expressions increased under stresses [13]. TGFβ1/pSmad3 signaling in fibroblasts was shown to be a crucial pathway of cardiac fibrosis [14], where expression of S100A4 and αSMA was upregulated by TGFβ1 via pSmad3 [15,16]. P21, which is involved in cell-cycle regulation and apoptotic signaling, was upregulated in fibroblasts by TNFα [17,18]. TNFα, a pro-inflammatory cytokine of one of the parameters for inflammation, was increased in fibrotic hearts [2,18]. Blockade of *TNFα* reportedly suppressed fibrosis in hypertrophic hearts [19]. Expression of αSMA, TNFα, and TGFβ1 in cardiac fibrosis induced by angiotensin II or TAC was shown to be inhibited in *Smad3* knock-out (*Smad3*KO) mice [13,20]. Since cardiac fibrosis and dysfunction in hypertrophic hearts remain major risk factors for cardiovascular morbidity and mortality [21], new molecular targets are required in the treatment of cardiac diseases.

*Dec1* (*Differentiated embryonic chondrocyte gene1*), also known as *BHLHE40*, *Stra13*, and *Sharp2*, is a basic helix–loop–helix (BHLH) transcriptional factor [22,23]. It can directly bind to E-boxes and Sp1 sites in the promoter region of the target genes, suppressing and promoting their transcriptions, respectively [22,23]. *Dec1* is associated with inflammation, circadian rhythm, hypoxia, and apoptosis [22,23,24]. It was proven that *Dec1* deficiency inhibits inflammation in periodontitis, obesity, and hypertension [25,26,27]. In our previous study, we showed that *Dec1* knock-out (*Dec1*KO) mice suppressed *TNFα*, *TGFβ1*, and *Interleukin-1 beta (IL-1β)* under *Porphyromonas gingivalis* infection [25]. Moreover, *Dec1* knockdown by small interfering RNA (siRNA) decreased pSmad3 induced by TGFβ1 in pancreatic cancer cells [28]. The deficiency of clock genes, such as *brain and muscle aryl hydrocarbon receptor nuclear translocator-like protein-1* (*Bmal1*), *Clock*, and *period 2* (*Per2*), showed impacts on the development of cardiac hypertrophy and fibrosis, as well as cardiac dysfunction [29,30,31]. In this study, to investigate the role of *Dec1* in cardiac fibrosis and its function in hypertrophic hearts, we examined cardiac fibrosis and associated molecules, as well as cardiac function in wild-type (WT) and *Dec1*KO mice treated with TAC.

## 2. Results

### 2.1. Dec1 Deficiency Protects Cardiac Function in Pressure Overload-Induced Cardiac Hypertrophy

**Figure 1:**
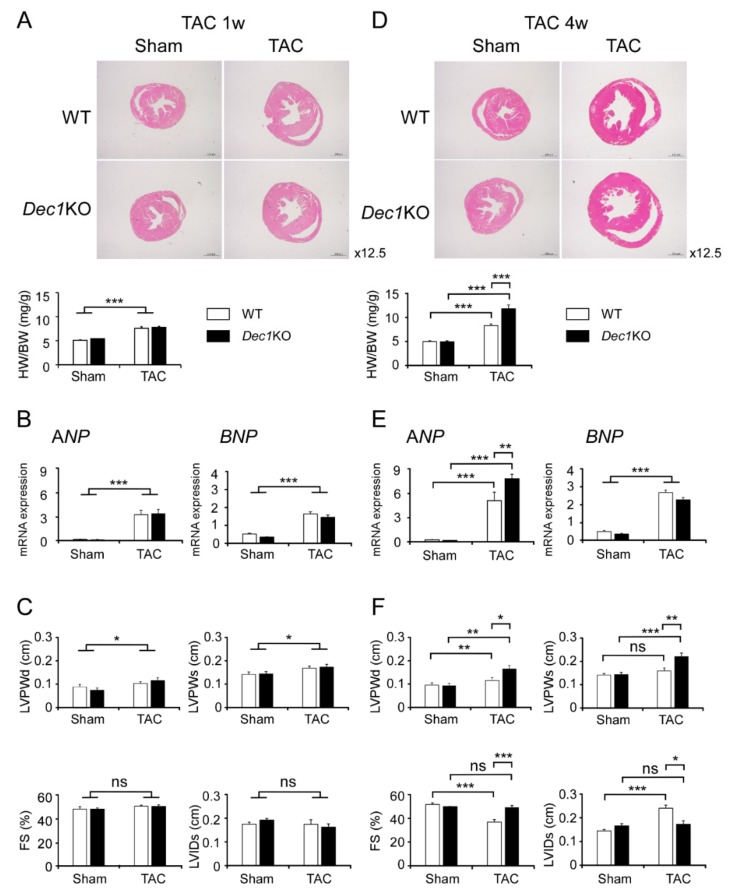
*Differentiated embryonic chondrocyte gene 1* (*Dec1*) deficiency protects cardiac function against transverse aortic constriction (TAC)-induced pressure overload. (**A**) Assessment of cardiac phenotype in wild-type (WT) and *Dec1* knock-out (*Dec1*KO) mice at one week (1w) after TAC (*n* = 11–13 mice per group) and sham treatment (*n* = 5–6 per group). Hematoxylin and eosin (H&E) staining of WT and *Dec1*KO hearts, magnification 12.5×. Heart weight/body weight (HW/BW). White box indicates WT and black box indicates *Dec1*KO. (**B**) The relative messenger RNA (mRNA) expression of the hypertrophy markers, *ANP* (atrial natriuretic peptide) and *BNP* (B-type natriuretic peptide), in WT and *Dec1*KO mice at one week after TAC and sham. (**C**) Echocardiographic analysis of WT and *Dec1*KO mice at one week after TAC and sham. FS: fractional shortening; LVIDs: left ventricular internal dimension at the end of systole; LVPWd and LVPWs: left ventricular posterior wall thickness at the end of diastole and at the end of systole, respectively. (**D**) Assessment of cardiac phenotype in WT and *Dec1*KO mice at four weeks (4w) after TAC and sham treatment (*n* = 8–11 per group). (**E**) The relative mRNA expression of the hypertrophy markers, *ANP* and *BNP*, in WT and *Dec1*KO mice at four weeks after TAC and sham treatment. (**F**) Echocardiographic analysis of WT and *Dec1*KO mice at four weeks after TAC and sham treatment. Multiple comparisons between sham and TAC groups were analyzed by two-way ANOVA with Tukey–Kramer post hoc test. Data are the means ± standard error of the mean (SEM). * *p* < 0.05, ** *p* < 0.01, *** *p* < 0.001; NS: not significant.

We firstly measured the ratio of heart weight to body weight (HW/BW) and examined cardiac function by echocardiogram for the intact *Dec1*KO mice. Intact *Dec1*KO mice did not show significant distinction in HW/BW ratio or fractional shortening (FS) compared with WT mice (Figure S1). To investigate the role of *Dec1* in pressure overload-induced cardiac dysfunction, we performed transverse aortic constriction (TAC) or sham surgery and evaluated cardiac function and morphology at one and four weeks after surgery. One week after TAC, cardiac hypertrophy was observed in both WT and *Dec1*KO mice. HW/BW ratio and left ventricular size assessed by hematoxylin and eosin (H&E) stain were increased in TAC-treated mice compared with sham-treated mice (Figure 1A). Additionally, the hypertrophic markers of *atrial natriuretic peptide (ANP)* and *B-type natriuretic peptide (BNP)* expressions increased in TAC-treated mice, although there were no significant differences in WT or *Dec1*KO mice (Figure 1B). Furthermore, the thickness of the left ventricular post wall at the end-diastole (LVPWd) and at the end-systole (LVPWs), as evaluated by echocardiogram, was higher in TAC-treated mice than in sham-treated mice (Figure 1C). On the other hand, cardiac function was preserved in both WT and *Dec1*KO mice, owing to either FS or left ventricular internal dimension at the end-systole (LVIDs) not being significantly different between groups (Figure 1C). Collectively, in our TAC model, both WT and *Dec1*KO mice showed cardiac hypertrophy with preserved cardiac function at one week after TAC. At four weeks after TAC, cardiac hypertrophy was severer in *Dec1*KO mice than WT mice. The increases in HW/BW ratio and left ventricle size were higher in TAC-treated *Dec1*KO mice than in WT mice (Figure 1D). The increase in *ANP* messenger RNA (mRNA) expression was higher in TAC-treated *Dec1*KO mice than in WT mice, whereas there was no significant change in *BNP* mRNA expression (Figure 1E). LVPWd and LVPWs were increased more in TAC-treated *Dec1*KO mice than in WT mice (Figure 1F). As expected, WT mice developed cardiac dysfunction with a decrease in FS and an increase in LVIDs due to TAC (Figure 1F). Nevertheless, interestingly, neither FS nor LVIDs was significantly altered in TAC-treated *Dec1*KO mice compared with sham-treated mice (Figure 1F). In addition, the decrease in FS and the increase in LVIDs due to TAC were suppressed in *Dec1*KO mice (Figure 1F). Taken together, these results indicate that cardiac function was preserved in *Dec1*KO mice until four weeks after TAC, suggesting that *Dec1* deficiency protects cardiac function in pressure overload-induced cardiac hypertrophy.

### 2.2. TAC Increases Dec1 Expression

**Figure 2:**
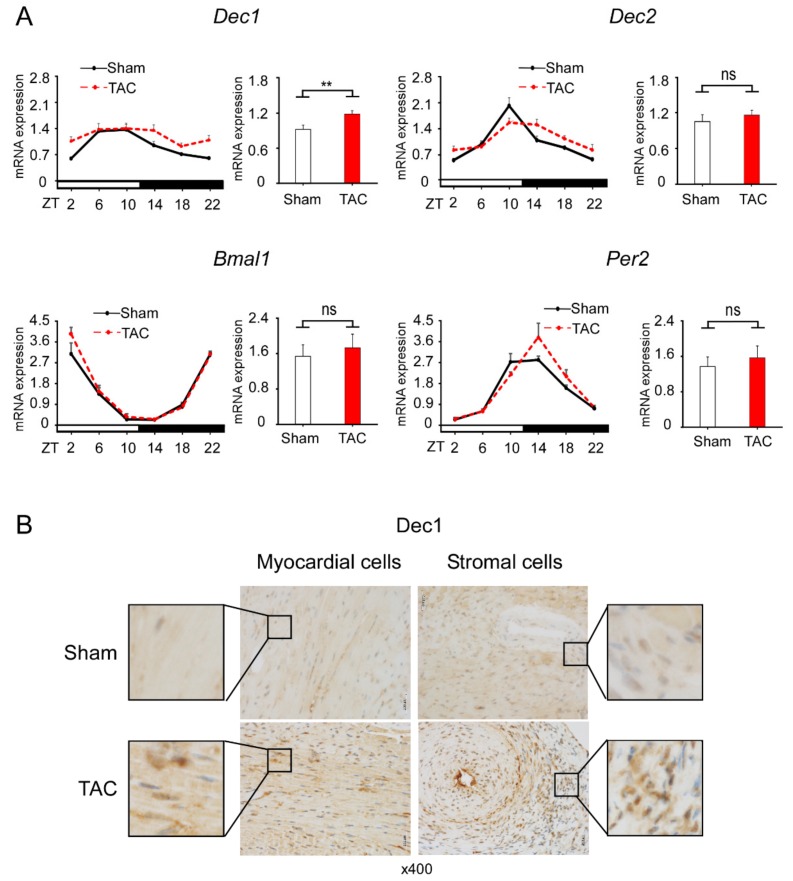
TAC increases *Dec1* expression. (**A**) The circadian expression of clock genes in WT mice treated with TAC (red dotted line) and sham treatment (black line). The mRNA levels of *Dec1*, *Dec2*, *brain and muscle aryl hydrocarbon receptor nuclear translocator-like protein-1* (*Bmal1*), and *period 2* (*Per2*) were analyzed by real-time PCR. Each right graph shows average of total mRNA expressions from zeitgeber time 2 (ZT2) to ZT22 in sham and TAC mice. The circadian expression of clock genes was assessed by analyzing one-way ANOVA. Multiple comparisons between sham and TAC groups were analyzed by two-way ANOVA with Tukey–Kramer post hoc test. Comparison of two groups was analyzed by a two-tailed Student’s *t*-test. The number of mice was four or five mice per group per time point. Data are the means ± SEM. ** *p* < 0.01; NS: not significant; ZT: zeitgeber time with light on at 8:00 a.m. (ZT0) and light off at 8:00 p.m. (ZT12). (**B**) Immunohistochemical detection of Dec1 in myocardial and stromal cells. Representative images of one WT heart treated with TAC and sham at four weeks. The black square shows representative large images, magnification 400×.

To examine whether TAC affects the expression of clock genes, we examined the expression levels of *Dec1*, *Dec2*, *Bmal1*, and *Per2* in TAC and sham-treated WT mice. All clock genes examined maintained the diurnal rhythms in the gene expression levels at four weeks after TAC and sham treatment (Figure 2A). Among these genes, the circadian expression of *Dec1* significantly increased after TAC, with the other genes not significantly changed compared to sham treatment. To investigate whether TAC also affects *Dec1* expression at the protein level, we performed immunohistochemistry. *Dec1* expression increased in both myocardial and stromal cells at one and four weeks after TAC treatment (Figure 2B and Figure S2), suggesting that TAC increases the Dec1 protein in the heart.

### 2.3. Dec1 Deficiency Suppresses TAC-Induced Cardiac Perivascular Fibrosis

**Figure 3:**
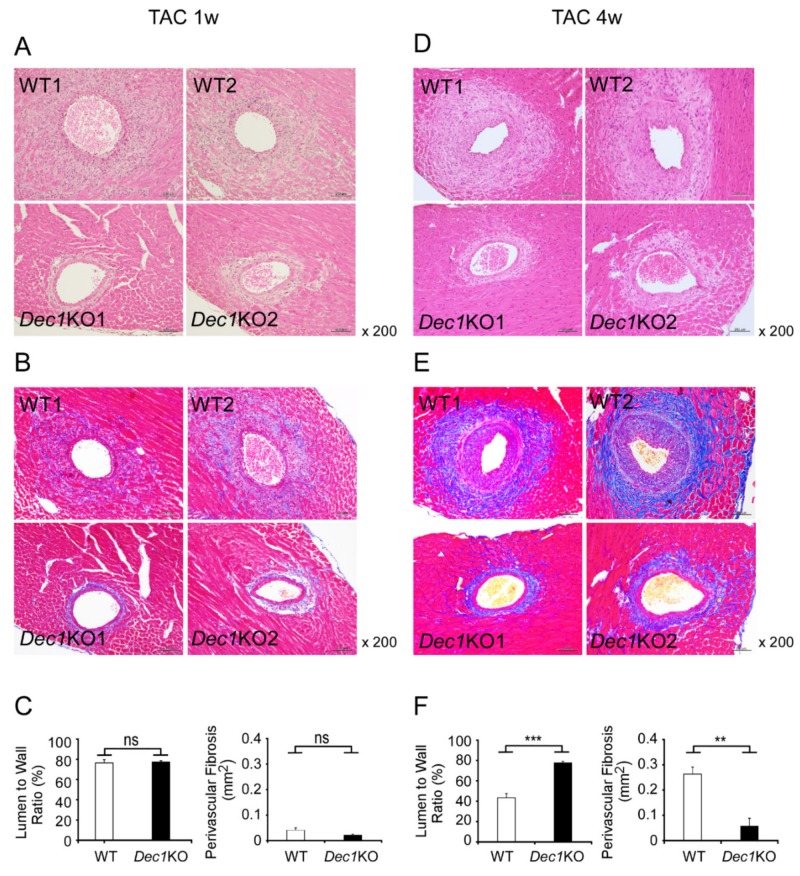
*Dec1* deficiency suppresses cardiac perivascular fibrosis induced by TAC. (**A**) Perivascular lesions of WT and *Dec1*KO hearts at one week after TAC. Representative images of two independent WT (WT1, WT2) and *Dec1*KO (*Dec1*KO1, *Dec1*KO2) mice are shown in H&E staining. (**B**) Masson’s trichrome (MT) staining at one week after TAC. Blue color shows fibrosis. (**C**) Quantification of lumen to wall perimeter at one week after TAC in H&E staining (left graph), and perivascular fibrosis area in MT staining (right graph). (**D**) Perivascular lesions of WT and *Dec1*KO hearts at four weeks after TAC. (**E**) MT staining at four weeks after TAC. Blue color shows fibrosis. (**F**) Quantification of lumen to wall perimeter and perivascular fibrosis area at four weeks after TAC. Original magnification 200× (**A**,**B**,**D**,**E**). Data are the means ± SEM of four mice and were analyzed by a two-tailed Student’s *t*-test. ** *p* < 0.01 *** *p* < 0.001; NS: not significant.

To explore the role of *Dec1* in cardiac fibrosis in pressure overload-induced cardiac hypertrophy, we assessed the development of cardiac fibrosis at one and four weeks after TAC in WT and *Dec1*KO mice. At one week after TAC, WT mice showed mild fibrosis (Figure 3A,B and Figure S3A). On the other hand, TAC-treated *Dec1*KO mice revealed little fibrosis (Figure 3A,B and Figure S3A). The thickness of the vascular wall was little affected in both WT and *Dec1*KO mice (Figure 3A). Consistently, the ratio of perimeter in lumen to wall was not significantly changed between WT and *Dec1*KO mice after TAC (Figure 3C). In addition, quantification of perivascular fibrosis by TAC showed a trend, but not a significant difference, to decrease in *Dec1*KO mice compared with in WT mice (*p* = 0.07). These results suggest that *Dec1* deficiency may inhibit perivascular fibrosis by TAC. At four weeks after TAC, severe perivascular fibrosis was observed in TAC-treated WT mice (Figure 3D,E and Figure S3B). Also, TAC-treated WT mice showed narrowed vascular lumen due to the thickened vascular wall (Figure 3D). In contrast, the size of vascular lumen was maintained in TAC-treated *Dec1*KO mice (Figure 3D). Furthermore, perivascular fibrosis by TAC was significantly suppressed in *Dec1*KO mice compared with in WT mice (Figure 3E,F). The ratio of perimeter in lumen to wall was also significantly higher in TAC-treated *Dec1*KO mice than in WT mice (Figure 3F). Taken together, these results indicate that *Dec1* deficiency suppresses cardiac perivascular fibrosis induced by TAC.

**Figure 4:**
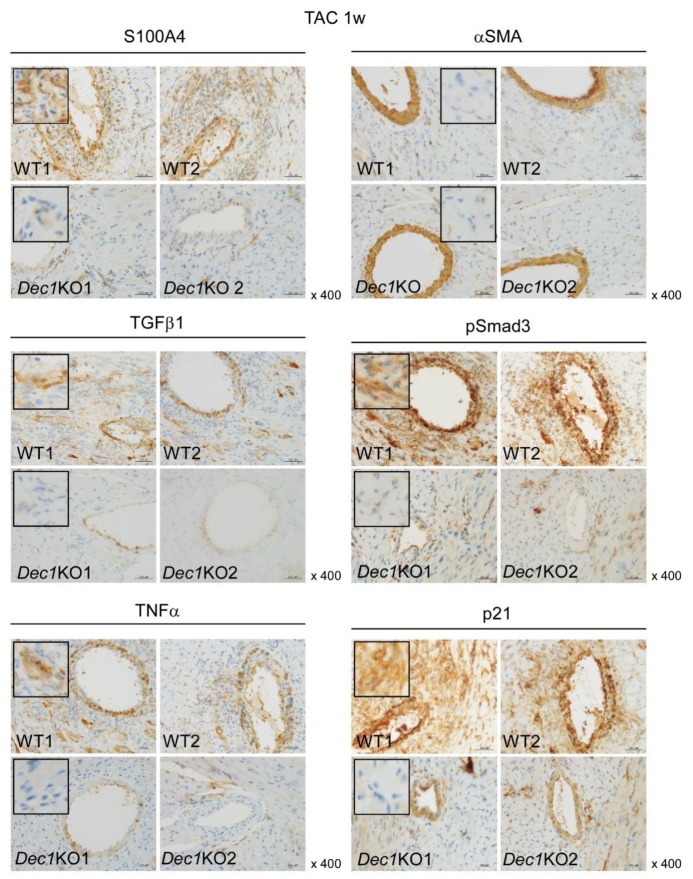
*Dec1* deficiency suppresses the expression of S100 calcium binding protein A4 (S100A4), transforming growth factor beta 1 (TGFβ1), phosphorylation of Smad family member 3 (pSmad3), tumor necrosis factor alpha (TNFα), and cyclin-interacting protein 1 (p21) at one week after TAC. Immunohistochemical detection of S100A4, alpha smooth muscle actin (αSMA), TGFβ1, pSmad3, TNFα, and p21 in cardiac perivascular lesions. Representative images of two independent WT (WT1, WT2) and *Dec1*KO (*Dec1*KO1, *Dec1*KO2) mice at one week after TAC. Magnification 400×. The black square was magnified from a representative lesion.

**Figure 5:**
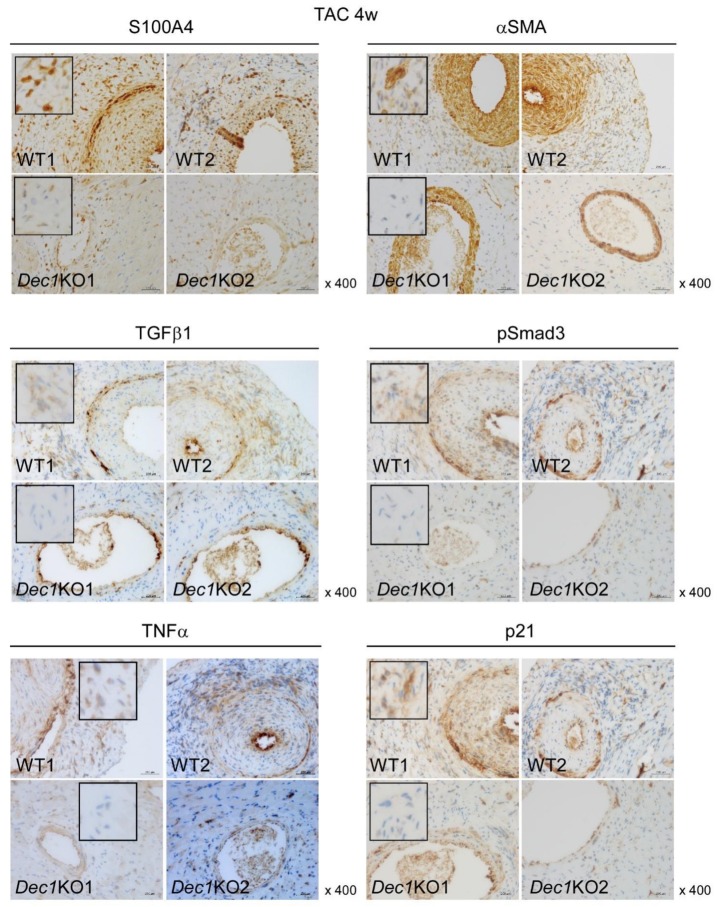
*Dec1* deficiency suppresses the expression of S100A4, TGFβ1, pSmad3, TNFα, and p21 at four weeks after TAC. Immunohistochemical detection of S100A4, αSMA, TGFβ1, pSmad3, TNFα, and p21 in cardiac perivascular lesions. Representative images of two independent WT (WT1, WT2) and *Dec1*KO (*Dec1*KO1, *Dec1*KO2) mice at four weeks after TAC. Magnification 400×. The black square was magnified from a representative lesion.

**Figure 6:**
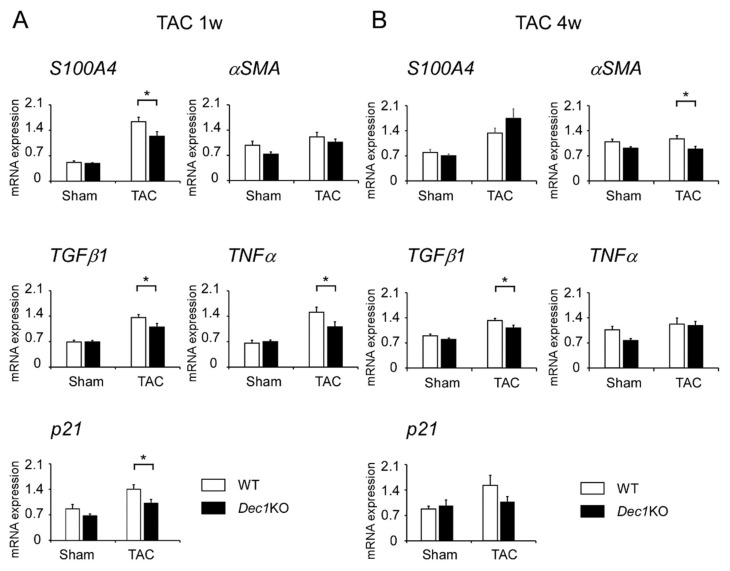
*Dec1* deficiency affects the mRNA expressions of *S100A4*, *αSMA*, *TGFβ1, TNFα*, and *p21* after TAC. (**A**) The relative mRNA expression of *S100A4*, *αSMA*, *TGFβ1*, *TNFα*, and *p21* in WT and *Dec1*KO hearts at one week after TAC (*n* = 11–13 mice per group) and sham treatment (*n* = 5–6 per group). The mean comparison between WT and *Dec1*KO mice after TAC and sham treatment was analyzed by a two-tailed Student’s *t*-test with 95% confident interval (CI). For TAC groups, the *p*-values were as follows: *S100A4*, 0.03 (95% CI: 0.0448, 0.7473); *TGFβ1*, 0.04 (95% CI: 0.0134, 0.5095); *TNFα*, 0.04 (95% CI: 0.0092, 0.7970); *p21*, 0.04 (95% CI: 0.0202, 0.7586). For sham groups, none were significant. The white box indicates WT, and the black box indicates *Dec1*KO. (**B**) Four weeks after operations (*n* = 8–11 mice per group). For TAC groups, the *p*-values were as follows: *αSMA*, 0.02 (95% CI: 0.0413, 0.5321); *TGFβ1*, 0.03 (95% CI: 0.0197, 0.3988). For sham groups, the *p*-value was as follows: *TNFα*, 0.01 (95% CI: 0.0656, 0.5337). Data are means ± SEM. * *p* < 0.05; NS: not significant.

Next, we examined expression levels of protein and mRNA that associated with cardiac fibrosis and inflammation such as S100A4, αSMA, TGFβ1, pSmad3, TNFα, and p21 by immunohistochemistry and real-time PCR. At one week, TAC-treated WT and *Dec1*KO mice increased expression of S100A4, TGFβ1, pSmad3, TNFα, and p21 compared to mice with sham treatment (Figure 4 and Figure 6A and Figure S4). However, the expression of αSMA was barely affected (Figure 4 and Figure 6A and Figure S4). On the other hand, the expression of S100A4, TGFβ1, pSmad3, TNFα, and p21 significantly decreased in TAC-treated *Dec1*KO mice compared with WT mice, but the expression of αSMA was barely affected (Figure 4 and Figure S6A). No obvious differences in these expressions were observed between WT and *Dec1*KO sham-treated mice (Figure 6A and Figure S4). At four weeks, TAC-treated WT and *Dec1*KO mice also increased the expression of S100A4, αSMA, TGFβ1, pSmad3, TNFα, and p21 compared to sham treatment (Figure 5 and Figure 6B and Figure S5). As expected, the increased expressions were significantly decreased in *Dec1*KO mice, observed by immunohistochemistry (Figure 5 and Figure S6B). In real-time PCR, at one week after TAC, the mRNA expression of *S100A4*, *TGFβ1*, *TNFα*, and *p21* was decreased in *Dec1*KO mice compared with that of WT (Figure 6A). The mRNA expression of *αSMA* was barely affected (Figure 6A). At four weeks after TAC, the mRNA expression of *αSMA* and *TGFβ1* decreased in *Dec1*KO mice compared with WT mice (Figure 6B). The mRNA expression of *S100A4*, *TNFα*, and *p21* was barely affected (Figure 6B). Collectively, these results suggest that *Dec1* deficiency is associated with cardiac fibrosis in pressure overload-induced cardiac hypertrophy.

### 2.4. Dec1 Expression Is Increased in Human Cardiac Hypertrophy and Myocardial Infarction

**Figure 7:**
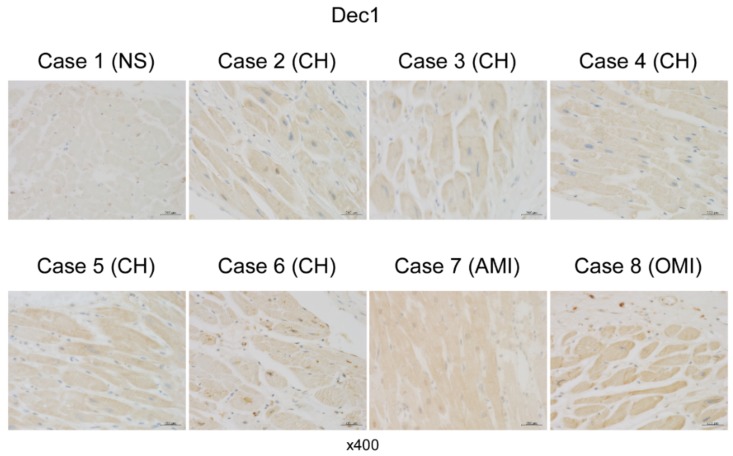
*Dec1* expression is increased in the myocardial cells of cardiac hypertrophy and myocardial infarction. Immunohistochemical detection of Dec1 in human cardiac diseases. Representative image of Dec1 immunoreactivities from case 1 to case 8. Case 1 (control) had no significant findings (NS). Cases 2 to 6 are cardiac hypertrophy (CH). Case 7 is acute myocardial infarction (AMI), and case 8 is an old myocardial infarction (OMI). Magnification 400×.

To explore *Dec1* expression in human cardiac diseases, we performed immunohistochemistry in the hearts of human autopsy cases: five cases of cardiac hypertrophy (CH), one case of acute myocardial infarction (AMI), and one case of old myocardial infarction (OMI). *Dec1* expression was slightly increased in myocardial cells of CH compared with those in the control (NS: not significant findings) and highly increased in those of AMI and OMI (Figure 7). These results suggest that *Dec1* may play an important role in human heart diseases.

## 3. Discussion

Cardiac fibrosis is a major factor in heart failure of hypertrophic hearts [4,5,6,12]. Thus, investigation of molecular targets in anti-fibrotic therapies for hypertrophic heart is necessary. In the current study, we demonstrated for the first time the role of *Dec1* in inhibiting cardiac fibrosis and improving the dysfunction in hypertrophic hearts induced by TAC. We found that TAC-treated *Dec1*KO mice have more severely hypertrophic hearts at four weeks than WT mice. In addition, TAC disrupted only *Dec1* circadian expression in the heart of WT mice. These results suggest that *Dec1* plays important roles in cardiac hypertrophy.

In previous papers, it was shown that TAC-induced hypoxia and inflammation in WT mice involves hypoxia-inducible factor 1 alpha (HIF-1α), TNFα, and TGFβ1/pSmad3 [4,9,10]. We showed that *Dec1* expression is induced by HIF-1α in oral cancer cells under hypoxia [32], by TNFα in breast cancer cells under apoptosis [33], and by TGFβ1/pSmad3 in epithelial–mesenchymal transition of pancreatic cancer cells [28]. An increase in *Dec1* expression by TAC may, therefore, depend on inductions of HIF-1α, TNFα, and TGFβ1/pSmad3. We also found an increase in *Dec1* expression in myocardial cells in hypertrophy and myocardial infarction of autopsy cases, while we could not observe a significant increase in interstitial fibroblasts. This discrepancy may depend on differential backgrounds between humans and mice. In humans, the patient may have not only cardiac diseases, but also other diseases. We propose that *Dec1* plays important roles not only in TAC in mice but also in human cardiac diseases.

It was reported that *Smad3*KO mice inhibit cardiac fibrosis, decreasing the expression of αSMA, TGFβ1, and TNFα [13,20]. Since we previously demonstrated that *Dec1* knockdown by siRNA decrease *TGFβ receptor I* and pSmad3 induced by TGFβ1 [28], we examined pSmad3 expression using *Dec1*KO mice in this study. As expression of αSMA, S100A4, TGFβ1, pSmad3, TNFα, and p21 was barely changed in sham-treated mice, *Dec1* may indirectly regulate the expression of αSMA, S100A4, TGFβ1, pSmad3, TNFα, and p21 via TGFβ1/pSmad3.

At four weeks after TAC, the extent of cardiac perivascular fibrosis decreased in *Dec1*KO mice, where perivascular fibrosis may depend on the levels of αSMA and S100A4 expressions. We also found that the expression of p21 apoptotic factor decreased in *Dec1*KO mice after TAC. This may imply that *Dec1* deficiency could inhibit TAC-induced apoptosis.

In real-time PCR, at one week after TAC, the mRNA expressions of *S100A4*, *TGFβ1*, *TNFα*, and *p21* decreased in *Dec1*KO mice. This result is compatible with the immunohistochemical data. We think that *Dec1* regulates these molecules at one week after TAC by transcriptional levels. At four weeks after TAC, the mRNA expressions of *αSMA* and *TGFβ1* decreased in *Dec1*KO mice. This result is also compatible with the immunohistochemical data. These findings suggest that *Dec1* regulates the expressions of *αSMA* and *TGFβ1* at four weeks after TAC by transcriptional levels. However, the mRNA expressions of *S100A4*, *TNFα*, and *p21* were barely affected, although they had significant changes in immunohistochemistry. Therefore, *Dec1* may regulate the expressions of S100A4, TNFα, and p21 at four weeks after TAC by post-transcriptional levels. Taken together, *Dec1* may regulate target factors at different periods by transcriptional or post-transcriptional mechanisms, respectively. In the future, we plan to determine how *Dec1* regulates these target factors at different periods.

It was proven that *Dec1*KO mice suppressed inflammation in periodontitis and obesity by decreasing inflammatory factors such as TNFα, IL-1β, and peroxisome proliferative active receptor gamma (PPARγ) [25,26]. In addition, it was shown that blood pressure was reduced in *Dec1*KO mice by regulating transcription of *ATPase Na^+^/K^+^ transporting subunit beta 1* (*Atp1b1*) directly, whereas anti-fibrotic *fibroblast growth factor 21* (*FGF21*) was increased in *Dec1*KO liver under oxidative stress [27,34]. *Dec1*KO mice, therefore, may have a protective role against stress. These results are compatible with our data, which showed that cardiac perivascular fibrosis and inflammation were suppressed in *Dec1*KO mice. We suggest that the decreased levels of cardiac perivascular fibrosis in *Dec1*KO mice induced by TAC contributed to improved cardiac dysfunction.

In conclusion, we demonstrated that *Dec1* deficiency suppressed cardiac perivascular fibrosis in hypertrophic hearts by TAC, resulting in preserved cardiac function. *Dec1* may be a new target in anti-fibrotic therapies for hypertrophic hearts.

## 4. Materials and Methods

### 4.1. Animals

All animal experiments were performed according to previously described protocols [24]. Six-to-eight-week-old male WT and *Dec1*KO of whole *Dec1* gene deletion mice with a C57BL/6 background were generated as previously described [34,35]. All mice were reared in 12-h/12-h light/dark cycles with lights on at 8:00 a.m.

### 4.2. Ethics Approval and Consent to Participate

This study was approved by the Wakayama Medical University Research Ethics Committee (Dec 15, 2015 Protocol No. 1715), and histological specimens were retrieved from our hospital archives.

### 4.3. Transverse Aortic Constriction (TAC)

TAC operation was performed as previously described [4]. Male mice (6–7 weeks old, 19–24 g body weight) were quickly anesthetized with an intraperitoneal injection of a mixture of ketamine (50 mg/kg) and xylazine (5 mg/kg). The anesthetized mice were placed in a supine position on a surgery board under stereomicroscopy (SZ-PT, Olympus, Japan) after trimming of chest. An endotracheal tube was inserted and then connected with a volume-cycled rodent ventilator (KN-58 SLA, Natsume Seisakusho Co. Ltd., Tokyo, Japan). The mice were ventilated with a tidal volume of 0.2 mL room air and a respiratory rate of 90 breaths/minute. During the surgical procedure, anesthesia was maintained at 1.5% isoflurane with 1.5–1.7 L/min of 100% O_2_. The chest cavity was exposed by thoracotomy with a small mid-line incision. Transverse aorta between the innominate and left common carotid arteries was tightly ligated with a 7-0 silk suture two times against a 27G blunt needle. The needle was promptly removed after ligation to yield a constriction 0.4 mm in diameter. The rib cage was closed with a 5-0 prolene suture and the skin was closed with a 6-0 prolene suture. The mice were placed in a warm cage at 37 °C until full recovery from anesthesia. Sham-treated mice underwent the same procedure but without ligation of transverse aorta. One set of treated WT and *Dec1*KO mice was retained for one week, while others were retained for four weeks before analysis.

### 4.4. Transthoracic Echocardiography

Briefly, the mice were anesthetized with an intraperitoneal injection of a mixture of ketamine (50 mg/kg) and xylazine (5 mg/kg) and placed in a supine position. Transthoracic echocardiography was performed by using a two-dimensional (2D) echocardiogram (Sonoscape Co. Ltd., Shenzhen, China) with a 12L MHz transducer (Sonoscape Co. Ltd.). An image of the heart was obtained in M-mode in the parasternal short-axis view of the mid-LV. LV diastolic posterior wall thickness (LVPWd), LV systolic posterior wall thickness (LVPWs), LV diastolic internal dimension (LVIDd), and LV systolic internal dimension (LVIDs) were measured from LV cross-sectional area. LV fractional shortening (FS%) was calculated using the formula, (LVIDd − LVIDs)/LVIDd × 100, as previously described [36].

### 4.5. Tissue Preparation

The WT and *Dec1*KO hearts were collected between 9:00 a.m. and 12:00 p.m. at one and four weeks after TAC and sham operations. For analyzing gene expression, the ventricular heart tissue was rapidly frozen in liquid nitrogen and then stored at −80 °C until RNA extraction. For histology, the heart tissue was fixed in 4% paraformaldehyde, processed, and embedded in paraffin. To analyze circadian rhythmic expression of clock genes, the ventricular hearts from WT mice treated by sham and TAC were obtained every four hours at four weeks after operation, beginning from 10:00 a.m. (zeitgeber time 2 (ZT2)). Histological specimens of autopsy cases were retrieved from our hospital’s archives according to guidelines of the Japan Society of Pathology. We defined no significant findings (NS) as previous described [18]. We selected one case of NS, five cases of cardiac hypertrophy (CH), one case of acute myocardial infarction (AMI), and one case of old myocardial infarction (OMI).

### 4.6. H&E and MT Staining

The 4-µm paraffinized sections of heart were cut and stained with H&E or Masson’s trichrome (MT) as previously described [18,24]. To analyze the ratio of perimeter in lumen to wall, different H&E stained vascular cross-sections were selected randomly for each mouse. The average of this ratio per mouse was calculated before analyzing results from two the TAC groups (*n* = 4 mice per group). For quantification of cardiac perivascular fibrosis, images of the MT stained cross-sectional vascular were captured with magnification at 200× as previously described [24]. The area of perivascular fibrosis was determined using ImageJ with a threshold from 50 to 200 (software from National Institutes of Health, MD, USA). The perivascular fibrosis of the mouse heart was averaged from its three vascular cross-sections. For statistical analysis, groups of four mice were considered as sets.

### 4.7. Immunohistochemistry

Immunohistochemical detection of Dec1, S100A4, αSMA, TGFβ1, pSmad3, TNFα, and p21 was performed by using a Discovery Auto-Stainer with automated protocols for the 4-µm paraffinized sections of heart tissues (Ventana Medical Systems, Inc., Tucson, AZ; Roche, Mannheim, Germany), as previously described [24].

### 4.8. Real-Time PCR

Total RNA of ventricular heart tissue was extracted with TRIzol RNA Isolation Reagents (Thermo Fisher Scientific Inc., Waltham, MA, USA). First-strand complementary DNA (cDNA) was synthesized using 250 ng of cDNA and the High-Capacity cDNA Reverse Transcription Kit (Thermo Fisher Scientific Inc. Real-time PCR was performed with 1× SYBR Premix Ex Tap II using a TP850 Thermal Cycle Dice Real-Time System (Takara Bio, Inc., Kusatsu, Japan), as previously described [36]. To analyze gene expression levels, the comparative Cycle threshold (CT) method was used, in which expression levels of target genes relative to *Glyceraldehyde-3-phosphate dehydrogenase (Gapdh)* were calculated. The primer sequences were designed as follows: *Dec1* forward (F) 5′–CATGAGAACACTCGGGACCT–3′, and reverse (R) 5′–CCACACGATGGAGATGAGTG–3′; *Dec2* forward (F) 5′–AAACCTGCGCCAAAGAAGT–3′, and reverse (R) 5′–CTGGGTGTCCAGCTCTCAA–3′; *Bmal1* forward (F) 5′–CCACCTCAGAGCCATTGATACA–3′, and reverse (R) 5′–GAGCAGGTTTAGTTCCACTTTGTCT–3′; *Per2* forward (F) 5′–TGTGCGATGATGATTCGTGA–3′, and reverse (R) 5′–GGTGAAGGTACGTTTGGTTTGC–3′; *ANP* forward (F) 5′–AGAGACGGCAGTGCTCTAGG–3′, and reverse 5′–AGCCCTCAGTTTGCTTTTCA–3′; *BNP* forward (F) 5′–CACCCAAAAAGAGTCCTTCG–3′, and reverse (R) 5′–GCCCAAAGCAGCTTGAGATA–3′; *TNFα* forward (F) 5′–AGCCGATGGGTTGTACCTTG–3′ and reverse (R) 5′–ATAGCAAATCGGCTGACGGT–3; *TGFβ1* forward (F) 5′–GTCAGACATTCGGGAAGCAG–3′ and reverse (R) 5′–TCCACATGTTGCTCCACACT–3; *αSMA* forward (F) 5′–AGATCACAGCCCTCGCA–3′ and reverse (R) 5′–AGAGTACTTGCGTTCTGGAG–3′; *S100A4* forward (F) 5′–TGCATTCCAGAAGGTGATGA–3′ and reverse (R) 5′–TGCAGGACAGGAAGACACAG–3; *p21* forward (F) 5′–GGACAAGAGGCCCAGTACTTC–3′ and reverse (R) 5′–AGAGTGCAAGACAGCGACAA–3′; *Gapdh* forward (F) 5′–CAAGGAGTAAGAAACCCTGGACC–3′ and reverse (R) 5′–CGAGTTGGGATAGGGCCTCT–3′.

### 4.9. Antibodies

The antibodies used in this study were as follows: Dec1 (1:200, rabbit polyclonal, NB100-1800; Novus Biologicals, CO, USA), αSMA (1:4000, mouse monoclonal, ab7817; Santa Cruz Biotechnology Inc., Santa Cruz, CA, USA), S100A4 (1:1000, rabbit polyclonal, ab27957; Abcam, Tokyo, Japan), TGFβ1 (1:100, goat polyclonal, sc-156-G, Santa Cruz Biotechnology Inc.), pSmad3 (1:1000, rabbit monoclonal, ab52903; Abcam), TNFα (1:300, rabbit polyclonal, NBP1-47581; Novus Biologicals), and p21 (1:200, rabbit polyclonal, sc-397; Santa Cruz Biotechnology Inc.).

### 4.10. Statistical Analysis

To compare data from two groups, an unpaired two-tailed Student’s *t*-test was used. For multiple comparisons of data from groups containing two variable factors, two-way ANOVA followed by Tukey–Kramer post hoc test was performed. Circadian rhythm of gene expression was analyzed by one-way ANOVA. All tests were performed using JMPpro software, version 13.0 (SAS Institute Inc., NC, USA). Data are shown as means ± standard error of the mean (SEM). A *p*-value <0.05 was considered to be statistically significant.

## Supplementary Materials

Supplementary Materials can be found at https://www.mdpi.com/1422-0067/20/19/4967/s1. Figure S1: Heart weight/Body weight ratio and fractional shortening in intact WT and *Dec1*KO mice at ZT2. HW/BW ratio in intact WT and *Dec1*KO mice. FS assessed by echocardiogram in intact WT and *Dec1*KO mice. Data are the mean ± SEM of five mice and were analyzed by a two-tailed Student’s t-test. NS: not significant.; Figure S2: TAC induced Dec1 expression at 1w after operation. (A) Immunohistochemical detection of Dec1 in myocardial and stromal cells. Representative images of two WT mice (WT1, WT2) treated by TAC and sham (sham1, sham2) at 1w. The black square shows representative large images, magnification 400×. (B) The relative mRNA expression of *Dec1* at 1w after TAC. Data are the mean ± SEM and analyzed by a two-tailed Student’s t-test. *** *p* < 0.001.; Figure S3: The observation of cardiac perivascular fibrosis at 1 and 4w after TAC. (A) H&E staining of perivascular lesions. Representative images of two independent WT (WT1, WT2) and *Dec1*KO (*Dec1*KO1, *Dec1*KO2) mice at 1w after TAC. (B) At 4w after TAC. Black arrow shows cardiac perivascular lesions. magnification 40×.; Figure S4: Immunohistochemical detection of S100A4, αSMA, TGFβ1, pSmad3, TNFα and p21 in WT and *Dec1*KO hearts at 1w after sham treatment. Magnification 400×.; Figure S5: Immunohistochemical detection of S100A4, αSMA, TGFβ1, pSmad3, TNFα and p21 in WT and *Dec1*KO hearts at 4w after sham treatment. Magnification 400×.; Figure S6: Quantification of S100A4, αSMA, TGFβ1, pSmad3, TNFα and p21 immunostaining in WT and *Dec1*KO hearts at 1 and 4w after TAC. (A) Positive staining for S100A4, αSMA, TGFβ1, pSmad3, TNFα and p21 in perivascular fibroblasts of WT and *Dec1*KO hearts at 1w after TAC. The cells were counted in eight to ten random microscopic fields of two independent samples at magnification 400×. (B) Positive staining for S100A4, αSMA, TGFβ1, pSmad3, TNFα and p21 in perivascular fibroblasts of WT and *Dec1*KO hearts at 4w after TAC. The cells were counted in six to eight random microscopic fields of three to four independent samples at magnification 400×. Data are the mean ± SEM and analyzed by a two-tailed Student’s t-test. * *p* < 0.05. ** *p* < 0.01. *** *p* < 0.001. NS: not significant.

## Abbreviations

*Dec1*	*Differentiated embryonic chondrocyte gene 1*
*Dec2*	*Differentiated embryonic chondrocyte gene 2*
TAC	Transverse aortic constriction
H&E	Hematoxylin and eosin
MT	Masson’s trichrome
*ANP*	*Atrial natriuretic peptide*
*BNP*	*B-type natriuretic peptide*
FS	Fractional shortening
LVPWd	Left ventricular posterior wall thickness at the end of diastole
LVPWs	Left ventricular posterior wall thickness at the end of systole
HW/BW	Heart weight/body weight
