# Supplementary material for: Dec1 Deficiency Suppresses Cardiac Perivascular Fibrosis Induced by Transverse Aortic Constriction
Source: Int J Mol Sci. 2019 Oct 8;20(19):4967. doi: 10.3390/ijms20194967 (PMC6802004; doi:10.3390/ijms20194967)
Supplement: Supplementary file 1 [file ijms-20-04967-s001.pdf]

Supplementary figures

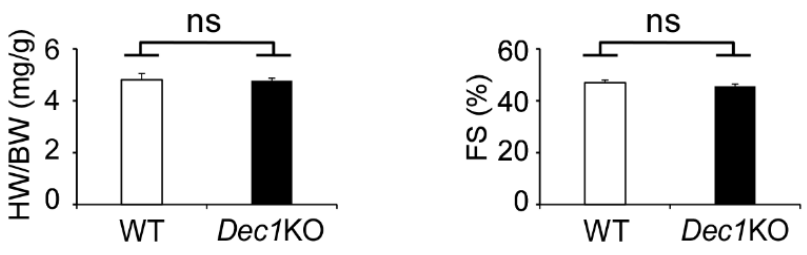

Figure S1. Heart weight/Body weight ratio and fractional shortening in intact WT and *Dec1*KO mice at ZT2. HW/BW ratio in intact WT and *Dec1*KO mice. FS assessed by echocardiogram in intact WT and *Dec1*KO mice. Data are the mean ± SEM of five mice and were analyzed by a two-tailed Student's t-test. NS: not significant.

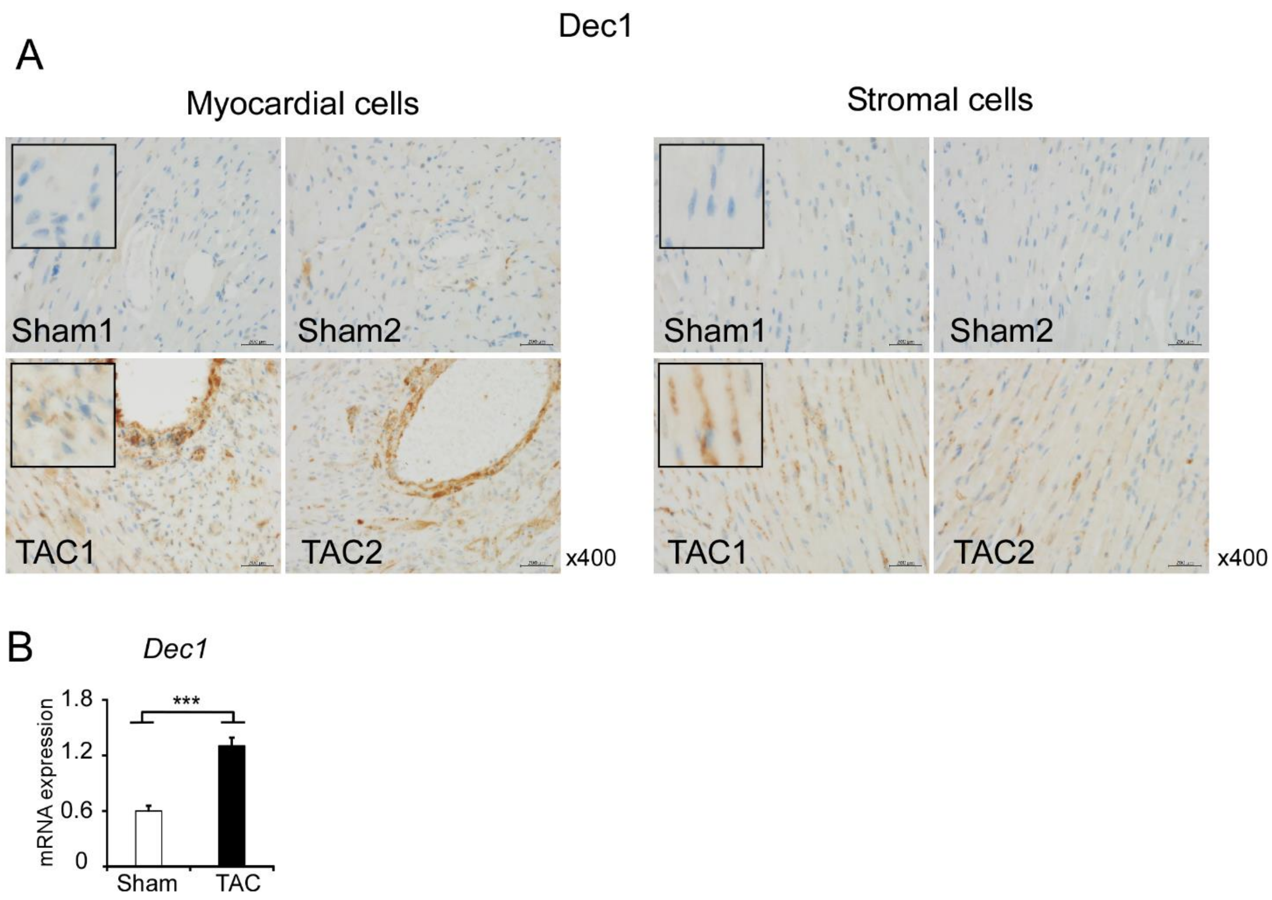

Figure S2. TAC induced Dec1 expression at 1w after operation. (**A**) Immunohistochemical detection of Dec1 in myocardial and stromal cells. Representative images of two WT mice (WT1, WT2) treated by TAC and sham (sham1, sham2) at 1w. The black square shows representative large images, magnification 400×. (**B**) The relative mRNA expression of *Dec1* at 1w after TAC. Data are the mean ± SEM and analyzed by a two-tailed Student's t-test. *** $p < 0.001$.

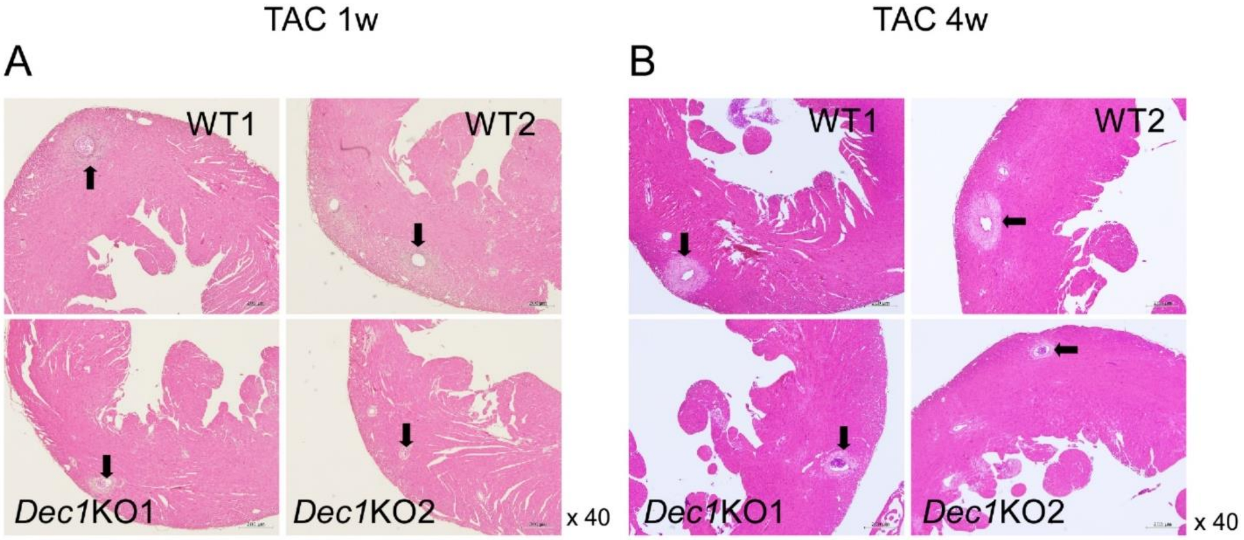

Figure S3. The observation of cardiac perivascular fibrosis at 1 and 4w after TAC. (**A**) H&E staining of perivascular lesions. Representative images of two independent WT (WT1, WT2) and *Dec1*KO (*Dec1*KO1, *Dec1*KO2) mice at 1w after TAC. (**B**) At 4w after TAC. Black arrow shows cardiac perivascular lesions. magnification 40×.

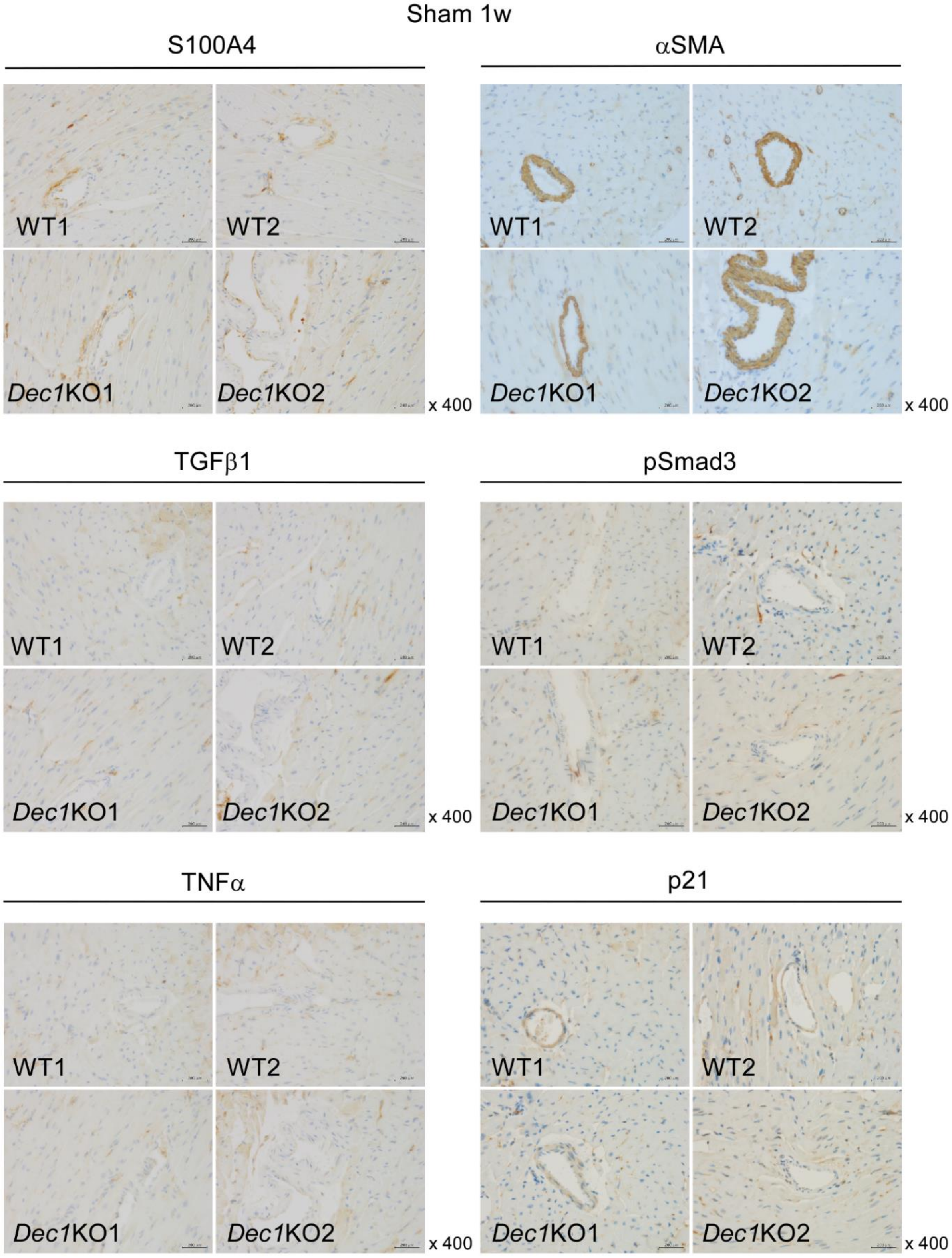

Figure S4. Immunohistochemical detection of S100A4, αSMA, TGFβ1, pSmad3, TNFα and p21 in WT and *Dec1*KO hearts at 1w after sham treatment. Magnification 400×.

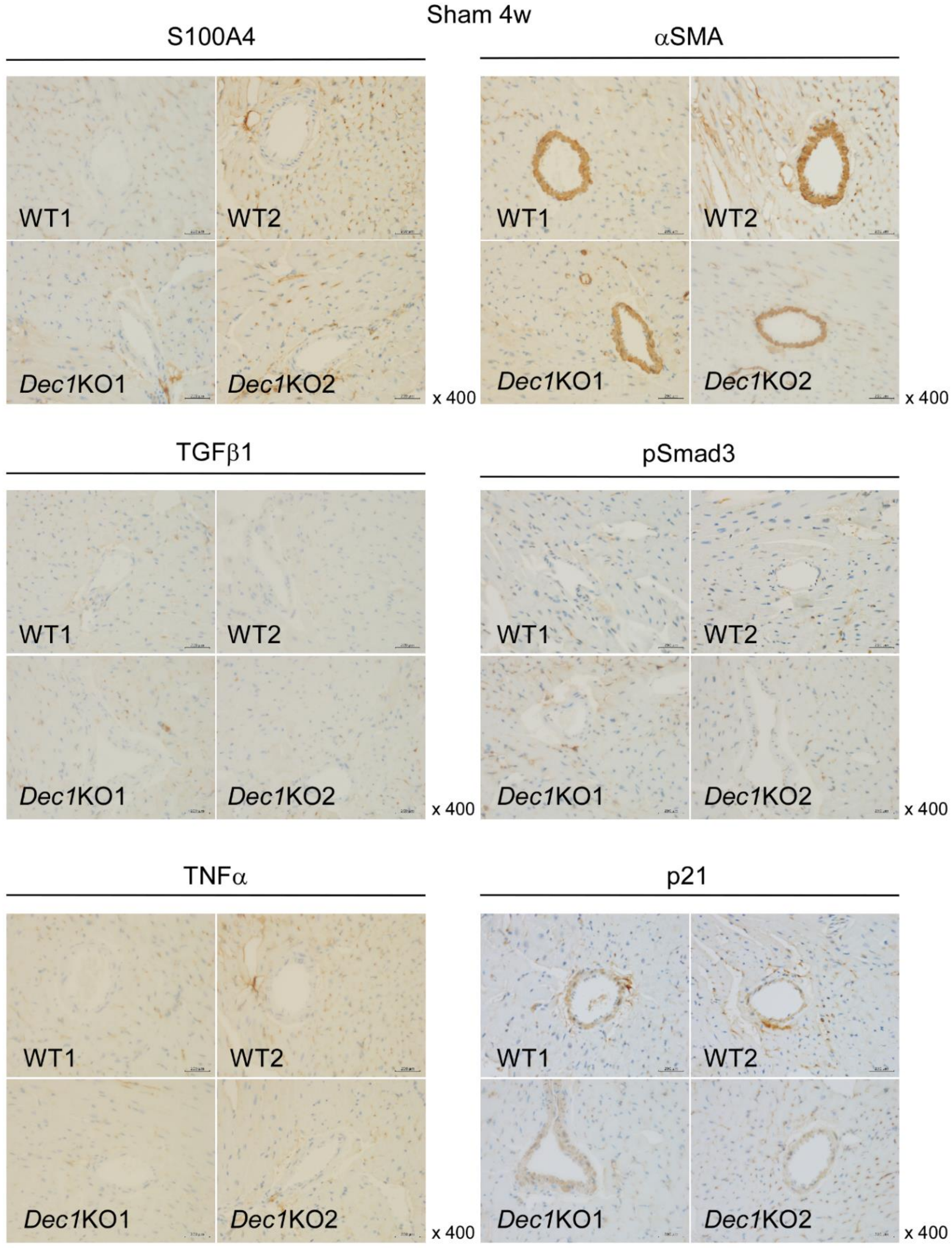

Figure S5. Immunohistochemical detection of S100A4, αSMA, TGFβ1, pSmad3, TNFα and p21 in WT and *Dec1*KO hearts at 4w after sham treatment. Magnification 400×.

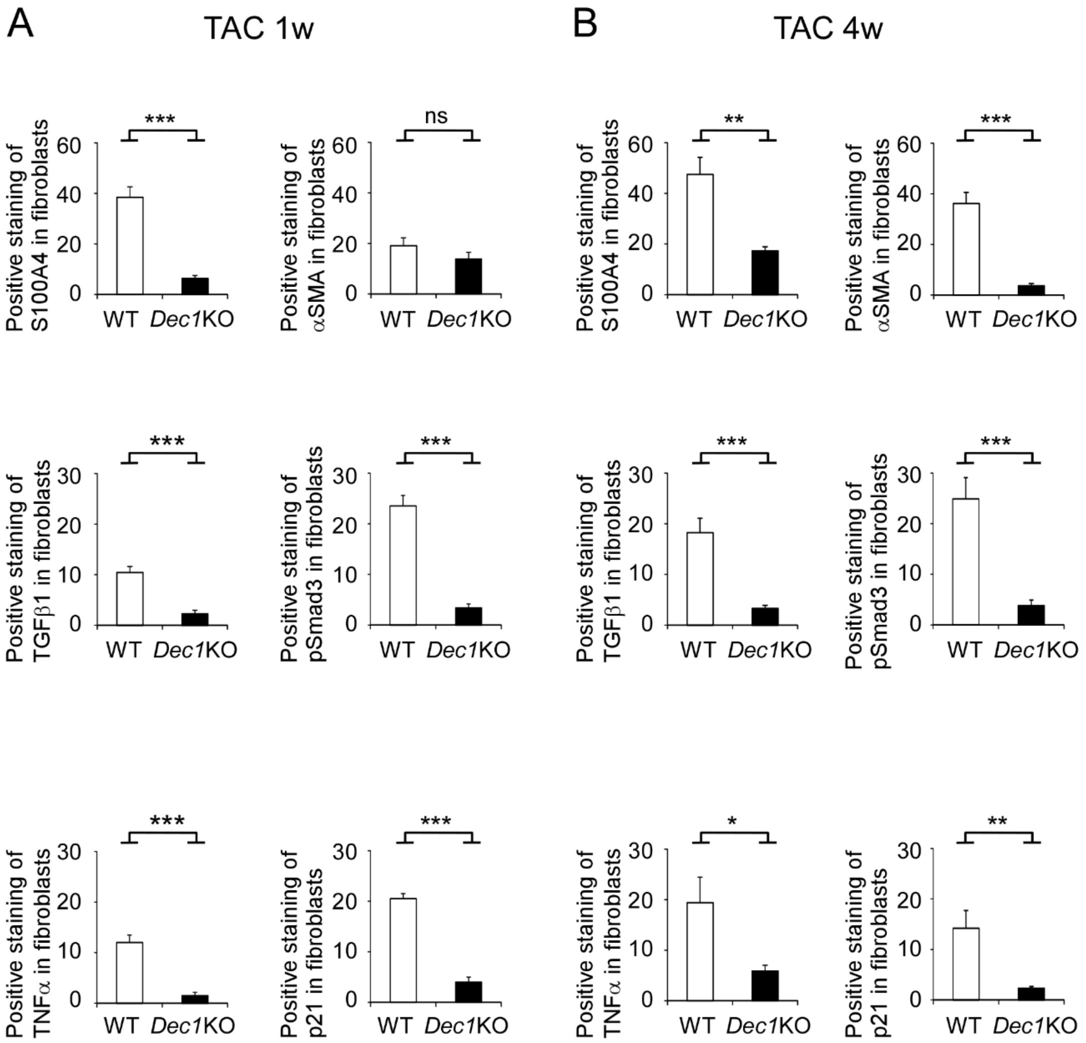

Figure S6. Quantification of S100A4, αSMA, TGFβ1, pSmad3, TNFα and p21 immunostaining in WT and *Dec1*KO hearts at 1 and 4w after TAC. (**A**) Positive staining for S100A4, αSMA, TGFβ1, pSmad3, TNFα and p21 in perivascular fibroblasts of WT and *Dec1*KO hearts at 1w after TAC. The cells were counted in eight to ten random microscopic fields of two independent samples at magnification 400×. (**B**) Positive staining for S100A4, αSMA, TGFβ1, pSmad3, TNFα and p21 in perivascular fibroblasts of WT and *Dec1*KO hearts at 4w after TAC. The cells were counted in six to eight random microscopic fields of three to four independent samples at magnification 400×. Data are the mean ± SEM and analyzed by a two-tailed Student's t-test. * $p < 0.05$. ** $p < 0.01$. *** $p < 0.001$. NS: not significant.
